# A review of the production, quality, and safety of traditionally fermented cereal‐based alcoholic beverages in Ethiopia

**DOI:** 10.1002/fsn3.4012

**Published:** 2024-02-13

**Authors:** Tolcha Techane Alemu, Chala G. Kuyu

**Affiliations:** ^1^ Department of Post‐Harvest Management Jimma University College of Agriculture and Veterinary Medicine Jimma Ethiopia

**Keywords:** cereal fermented beverages, quality, safety, traditional beverages

## Abstract

In Ethiopia, a diverse array of cereal‐based alcoholic beverages is being prepared and consumed. These traditional fermented drinks are distinct to specific regions and are prepared by locals using locally available raw materials according to cultural traditions. Notable among these are *Tella*, *Areki*, *Keribo*, *Borde*, and *Shamita*, renowned for their nutritional benefits and their role in ensuring food security. This paper explores existing literature regarding the production, quality, and safety of traditional cereal‐based alcoholic beverages in Ethiopia. Despite the widespread consumption of these beverages, they have yet to be commercialized, mainly due to their perceived low quality. The uncommercialized processes and products demand more attention, particularly in light of the country's inflationary pressures. Additionally, these traditional fermented beverages significantly enhance health due to the presence of bioactive compounds and their nutritional value. Standardizing and modernizing production methods by integrating scientific knowledge, such as optimizing fermentation practices, is essential to fully capitalize on these traditional beverages. Equipping local producers with this knowledge can facilitate the transition to larger scale production. Furthermore, continued research is essential to maintaining overall quality and safety standards. Therefore, it is crucial to concentrate on enhancing the nutritional value and quality of traditional cereal‐based beverages in the future. By illuminating these aspects, this review aims to enhance understanding of the traditional Ethiopian alcoholic beverage industry and its potential for elevating quality and safety standards. Moreover, the review explores these beverages' cultural significance, consumption patterns, and associated health risks.

## Graphical abstract

In Ethiopia, a diverse array of cereal‐based alcoholic beverages is being prepared and consumed. These traditional fermented drinks are distinct to specific regions and are pretended by locals using locally available raw materials by cultural traditions. Notable among these are Tella, Areki, Keribo, Borde, and Shamita, renowned for their nutritional benefits and their role in ensuring food security. Standardizing and modernizing production methods by integrating scientific knowledge, such as optimizing fermentation practices, is essential to capitalize fully on these traditional beverages.

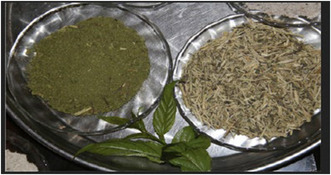

## 1 INTRODUCTION

Traditional fermented cereal beverages have been fundamental staples in various ancient civilizations. The methods for preparing these traditional beverages have been passed down from generation to generation (Kitessa, Bacha, Tola, Murimi, Smith, & Gershe, 2022; Kitessa, Bacha, Tola, Murimi, Gershe, & Guta, 2022; Muhialdin et al., 2022). During the medieval era, both alcoholic and non‐alcoholic fermented beverages were produced. The inclusion of cereals in the human diet marked a significant development, requiring remarkable technical and culinary skills to transform grains into staple foods. People have been searching for more affordable beverages that balance their economy, leading to fermentation primarily for safety reasons (Kitessa, Bacha, Tola, Murimi, Gershe, & Guta, 2022; Kitessa, Bacha, Tola, Murimi, Smith, & Gershe, 2022).

Fermentation extends the shelf life by reducing pH, increasing acidity, and minimizing contamination by microorganisms, but it also plays a crucial role in improving sensory properties such as texture, taste, and aroma. Additionally, it enhances the nutritional values and digestibility of foods while reducing the antinutritional contents present in cereals. Fermentation also improves the bioaccessibility and bioavailability of nutrients from different crops. According to Tekle et al. (2019), cereal‐based beverages have been produced and consumed for an extended period, with origins dating back to around 6000 BC. These traditional methods have led to fermented beverages constituting between 20 and 40% of individual diets and accounting for approximately one‐third of global food consumption (Chileshe, Talsma, et al., 2020; Chileshe, van den Heuvel, et al., 2020).

Countries around the world have their own traditional alcoholic beverages made from a variety of cereals. Ethiopia, in particular, is known for its wide range of traditional fermented beverages, such as *Tella*, *Shamita*, *Borde*, *Keribo*, and *Areki*, commonly enjoyed at social gatherings (wedding ceremony system, Iqubi) and during holidays (Nemo & Bacha, 2020). These beverages not only play a cultural role but also offer health benefits. The raw materials used for their production and flavor, as well the seasonal availability, make these beverages different from those from other world countries. Cereal beverages can be considered functional foods because they provide health benefits beyond essential nutrition. It is worth noting that yeast and *Lactobacillus* species are key microorganisms involved in the production of *Tella*. Sharma and Yaiphathoi (2020) reported that different microorganisms (bacteria, yeast, and molds) are involved in ethnic alcoholic beverage fermentation, and these microorganisms are naturally probiotic and important for human health. According to Nemo and Bacha's report (2020), fermentation enhances cereal beverages' nutritional, sensory, and functional qualities. Consuming these traditional fermented beverages with meals supports digestion and promotes the growth of beneficial bacteria. It is essential to recognize that most of Ethiopia's cereal‐based beverages are localized and have been integral to food and culture for generations. Hotessa and Robe (2020) emphasized that Ethiopian locally fermented beverages result from acid‐alcohol fermentation and are typically made from cereals such as barley, maize, and wheat. Barley, in particular, is gaining renewed interest due to its nutraceutical benefits. Its properties are known to offer protection against degenerative diseases, including diabetes, obesity, hypertension, and colon inflammation, which are often associated with unhealthy diets and lifestyles (Farag et al., 2022). This is largely attributed to its high beta‐glucan content, a type of dietary fiber. Furthermore, barley is rich in protein, minerals, vitamins, and carbohydrates, making it an excellent food supplement (Farag et al., 2022).

The preparation of cereal‐based fermented beverages in Ethiopia is typically a straightforward process without the need for complex methods. However, despite this simplicity, there needs to be more scientific knowledge regarding their production and impact on these traditional beverages' nutritional quality and safety. It is crucial to document this indigenous knowledge because the traditional art of brewing cereal beverages is transmitted orally and through experimentation from one generation to the next, without any formal procedures for preservation or production.

In many cases, non‐fermented beverages' nutritional value and shelf life are relatively lower than those of fermented beverages. Fermented beverages hold significant importance in society, with a high protein contribution, as they involve microbial processes that benefit human health (De la Bastida et al., 2023). Fermented beverages contain bioactive substances that can help reduce or inhibit allergies and diseases. Research by Kuyu and Bereka (2019) has highlighted fermentation as a key technique for producing quality food products, playing a crucial role in enhancing beverages by preserving sensory and nutritional qualities such as protein and vitamins.

Currently, the price of other beverages like beer has risen in various parts of Ethiopia, while traditional beverages like Tella, often referred to as Ethiopian beer, and others prepared in non‐standardized ways using earthen vessels may need better quality (Wolkers‐Rooijackers et al., 2018). The need for standardized and scientific procedures for preparing traditional beverages at the local level remains a significant challenge. Therefore, this review paper aims to consolidate studies conducted by numerous researchers on the production, quality, and safety of traditionally fermented cereal‐based alcoholic beverages consumed in Ethiopia.

## 2 METHODOLOGY

This review paper incorporated published results from both qualitative and quantitative data on the production, quality, and safety of traditionally fermented cereal‐based alcoholic beverages in Ethiopia. Data for this review were gathered from various internet sources, including the Essential Electronic Agriculture Library (TEEAL), Health InterNetwork Access to Research Initiative (HINARI), and 2023 Google Scholar databases. In total, fewer than sixty (60) published papers were collected from these specified sources and included in this review. All these sources were utilized to enhance the understanding of the production, quality, and safety of traditionally fermented cereal‐based alcoholic beverages in Ethiopia.

## 3 CEREAL‐BASED ALCOHOLIC BEVERAGES IN ETHIOPIA AND THEIR PRODUCTION

In Ethiopia, various traditional beverages are produced locally, often for home consumption or sale. Mothers employ various techniques to produce these beverages from different cereals. Popular traditional fermented alcoholic beverages in Ethiopia include *Tella*, *Borde*, *Keribo*, *Shamita*, and *Areki* (Bereka et al., 2022; Negasi et al., 2017). Alcoholic beverages are an integral part of the human dietary culture and are closely connected to human history. The production and consumption of alcoholic beverages from cereals serve as a way to enhance both the social and nutritional significance of food for people. Fermented food has been a traditional part of human consumption, with as many varieties as there are civilizations. However, preparing locally fermented beverages at the household level often results in poor quality and inconsistencies. Food Science and Technology has evolved from creating nutritious foods to developing foods with health‐enhancing properties, such as traditional cereal‐based beverages (Phiri et al., 2020). Some of the Ethiopian cereal‐based traditional fermented alcoholic beverages listed in Table 1

**TABLE 1:** Cereal‐based Ethiopian traditional fermented alcoholic beverages.

Order	Beverages	Raw materials/ingredients/	Consumption places
1	Tella	Barley (*Hordeum vulgare* L.), Wheat (*Triticum aestivum* L.), Maize (*Zea mays* L.), Finger millet (*Eleusine coracana* L.), Sorghum (*Sorghum bicolor* L.), Teff (*Eragrostis tef* L.), Gesho (R. prinoides)	Amhara, Oromia, Tigray,SNNP, Addis Ababa
2	*Borde*	Maize (Z. mays), Barley (H. Vulgare), wheat (T. aestivum), finger millet (E. coracana), sorghum (S. bicolor)	South nation nationality of peoples (SNNP)
3	*Shamita*	Roasted Barley (H. Vulgare) flour, salt, Linseed (*Linum usitatissimum* L.) flour, Chili pepper (capsicum annuum)	SNNP, Addis Ababa
4	Korefe	Malted and non‐malted barley (H. vulgare), Gesho (R. prinoides)	Amhara
5	Keribo	Barley (H. vulgare), sugar, baker yeast (Saccharomyces cerevisiae)	Oromia, Amhara, Addis Ababa
6	Cheka	Sorghum (*S. bicolor*), maize (*Z. mays*), finger millet (*E. coracana*), vegetables, root of taro (*Colocasia esculenta* L.)	SNNP
7	Areki	Barley (H. vulgare), Gesho (R. prinoides), sorghum	Amhara, Oromia, Tigray, SNNP, Addis Ababa

*Note*: Source: Fentie et al. (2020).

### 3.1 Tella

*Tella* is a ubiquitous and popular beverage in Ethiopia, often called traditional Ethiopian beer (Tesfaye, 2022). *Tella* is a fermented traditional beverage with a color ranging from grayish‐white to brown, depending on the degree of roasting, and it is the most widely brewed and consumed alcoholic beverage in almost every household (Fentie et al., 2020; Talema & Nega, 2022). The intensity of specific processing steps determines the beverage's color during preparation. *Tella* is prepared in Ethiopia from cereals such as barley, wheat, maize, millet, sorghum, and teff.

Research by Birhanu et al. (2021) revealed that *Tella* contains a low alcohol content in the range of 2%–4% or g/100 mL, making it a crucial source of nutrition in rural areas due to its turbidity with suspensions and significant presence of lactic acid bacteria. They also reported that *Tella* is rich in calories and minerals, particularly zinc, calcium, magnesium, and iron. However, Tekle et al. (2019) reported a higher content of alcohol ranging from 4 to 6% for filtered.

#### 3.1.1 Tella preparation

The method for making *Tella* varies across different ethnic groups in Ethiopia and is influenced by local customs and economic conditions (Lee et al., 2015). However, the basic processing steps are generally similar. Women predominantly prepare *Tella* beverages, as it is relatively rare for men to possess the skill for their preparation. According to a study by Getaye et al. (2018), the production of *Tella* beverage involves combining 1 kg of ground Gesho, 0.5 kg of malt, 15 kg of grains, 5 kg of bread (locally called Kita), and 10 kg of roasted and ground grain powder (Enkuro) with 30 L of pure water.

The key steps for *Tella* preparation are as follows: (1) Cleaning the container and washing it with water, then cleaning with Grawa leaves (*Vernonia amygdalina*); (2) Fumigating the container with the smoke of burning weyra (*Olea europaea*) or tin jute for 10–15 min to eliminate microorganisms and impart a unique flavor to the Tella; (3) Grinding bikil, prepared from dried germinated cereal like barley, to create malt, then moistening the cereal in a container and allowing it to germinate for 3 days before being sun‐dried to remove moisture; (4) Preparing Gesho plant (Rhamnus prinoides) leaves to impart a bitter flavor to the *Tella* and provide antibacterial effects; (5) Grinding barley into flour to make an unleavened bread called kita facilitates the process during enkuro, reducing its size and aiding in alcohol conversion; (6) Breaking the bread (kita) into small pieces; (7) Finally, roasting and grinding cleaned barley into flour (Enkuro), with the degree of roasting determining the color of the *Tella*.

#### 3.1.2 Cleaning agents in *Tella* preparation

Grawa (Vernonia amygdalina), also known as the bitter leaf, is used as a cleaning agent for *Tella* containers and holds medicinal properties. Additionally, Weira (*Olea europaea*), a sub‐species of olive, contains various chemicals that act as a multi‐chemical defense against insect and microbial attacks. In Ethiopia, Weira is commonly used to smoke fermentation in traditional drink containers, including those for *Tella*, water, milk, and milk products. After the container is cleaned and prepared, it is inverted over smoking wood fragments of weyra for 10–15 min, removing microorganisms sensitive to wood smoke and adding flavor to *Tella* (Wedajo Lemi, 2020). Weyra provides flavor for *Tella*, can kill bacteria, and extends *Tella's* shelf life.

#### 3.1.3 Ingredients used during the fermentation of *Tella*

**FIGURE 1:**
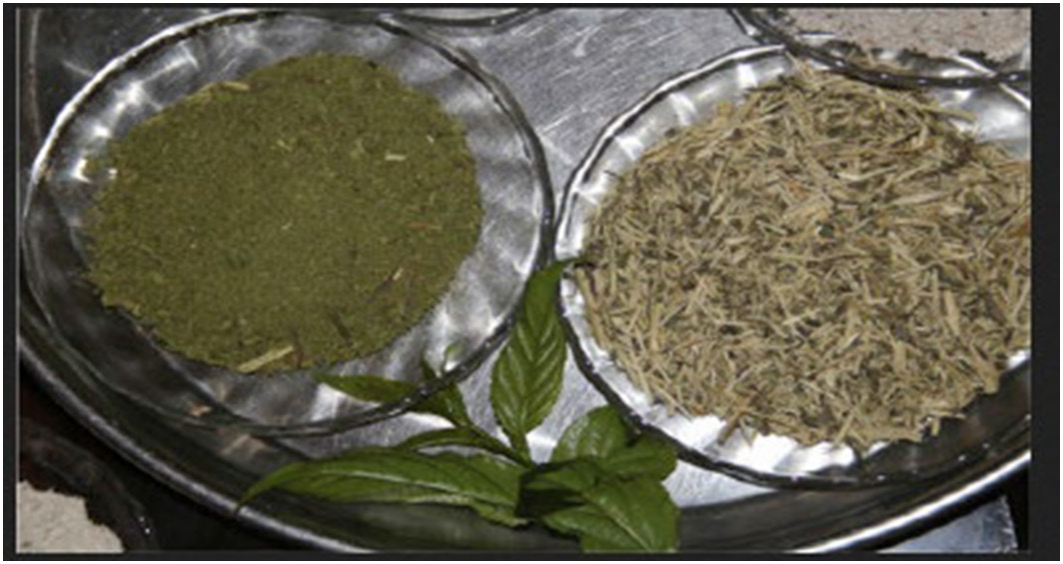
Gesho powder form. Left, powdered leaves; right, suitable stem shreds of gesho.

Gesho (Rhamnus prinoides) is a dense shrub resembling a small tree with spineless and evergreen leaves. Research has identified 20 secondary metabolites from Gesho plant leaves, stems, and fruits. Studies have shown that Gesho is a unique plant metabolite with bittering characteristics assumed to impart a bitter taste to the alcoholic beverage *Tella*. Both the leaves and stem are shredded, producing powder leaves, leaves, and stem shreds of Gesho (Lee et al., 2015), as shown in Figure 1 from the left powdered leaves and at the right side suitable stem shreds of Gesho.

**FIGURE 2:**
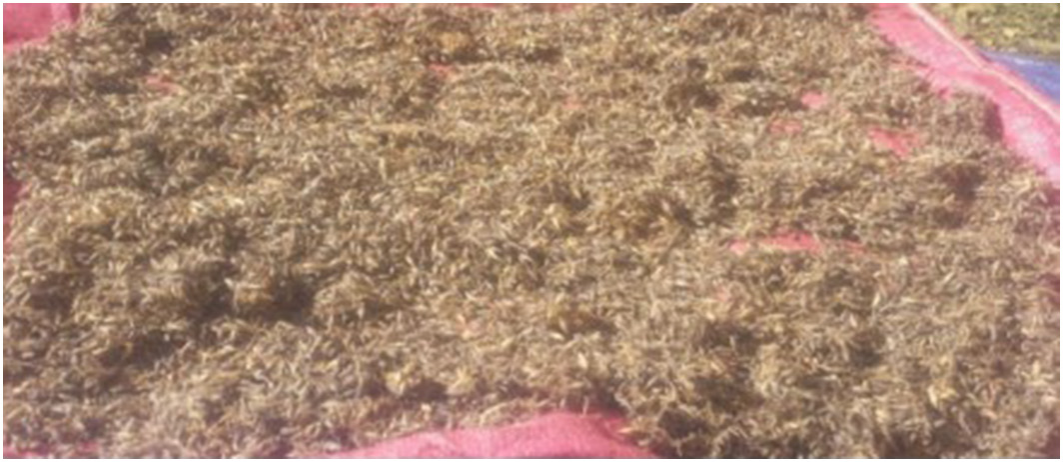
Malt from barley.

Malt is another major ingredient that serves as the primary source of yeast in *Tella* preparation (Sohrabvandi et al., 2020). Typically, malt is prepared from malted cereals, such as wheat or barley. The process involves soaking the cereal in water for around 2 days to initiate germination. The following day, the water is removed, and the cereal is placed in a container for another day to allow germination (Sohrabvandi et al., 2020; Wedajo Lemi, 2020). The prepared malt from barley resembles the image displayed in Figure 2 (Minbale, 2021).

#### 3.1.4 Tella fermentation phases

The fermentation of *Tella* is characterized by four distinct phases, as highlighted in various reports (Getaye et al., 2018; Sohrabvandi et al., 2020; Tafese et al., 2022). Initially, the first phase involves mixing ingredients, followed by successive additions of carbohydrate materials in the second and third phases. The primary carbohydrate materials are Malt, Kita, and Enkuro. The final phase is marked by acidification, which is generally considered undesirable.

The third phase and the beginning of the fourth phase are when alcohol production starts. Many reports have indicated that the color of *Tella* is directly influenced by the extent of heat treatment given to the Asharo (roasted barley) and the degree of steaming for the Enkuro (roasted barley steamed after grinding), a factor primarily determined by the individual preparing the *Tella*. Achieving the optimal ratio of ingredients is crucial for obtaining high‐quality *Tella*.

The fermenting organisms of *Tella* consist of Saccharomyces *cerevisiae* and *Lactobacillus* spp. The increase in ethanol content is directly related to the growth of the yeast population and a decrease in sugar and total carbohydrates. Generally, good‐quality *Tella* exhibits a final ethanol content ranging from 2–8% and a pH of 4–5 (Alemayehu, 2018; Megersa, 2015).

#### 3.1.5 Quality and safety of Tella

The quality of *Tella* relies on the quality and safety of the raw materials used and the method of preparation. To ensure the overall quality and safety of *Tella*, a systematic approach such as a hazard analysis critical control point, which is used for the identification, evaluation, and control of hazards, is required. *Tella* typically has a shelf life of 5–7 days at room temperature (Tekle et al., 2019). A study by Alemayehu (2018) suggests that better *Tella* contains 4.8% ethanol and a pH of 3.28. Critical control points (CCPs) are operational steps in a manufacturing process that, if not controlled, could result in injury or pose a risk to consumers (Mulaw et al., 2019). These steps are implemented after the fermentation process to maintain its shelf life.

**FIGURE 3:**
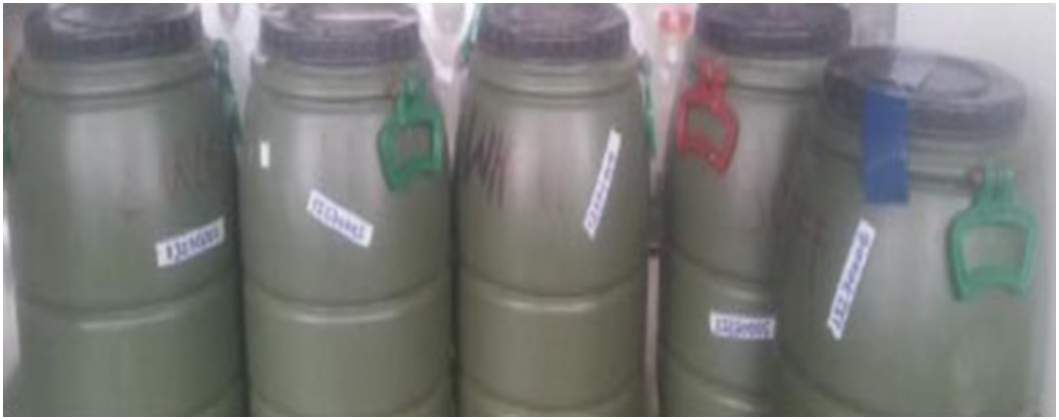
Traditionally prepared *Tella*.

Some critical control points at different stages of *Tella* preparation are as follows: (1) Quality of the raw materials: The raw materials for *Tella* preparation include gesho, water, and barley (or other cereals). Prior to malting, it is crucial to ensure that the barley is free of potential risks, as residues from pesticides and herbicides used on malting barley can persist into the finished malt, impacting the production process and product quality. (2) Malting process: Malted barley is allowed to germinate in an unsupervised environment with fluctuating moisture and temperature levels. (3) Kilning: The survival of enzymes used in the mashing process and the malt color depend on the kiln temperature. The malt is dried to an unknown extent by sunlight, and adjustments are not made to time, temperature, and moisture content to regulate the development of color and flavor. (4) Fermentation: In most cases, top fermentation at 18–22°C occurs by Saccharomyces *cerevisiae*, while bottom‐fermenting with a temperature range of 7–15°C is done by Saccharomyces *uvarum* (Meier‐Dörnberg et al., 2017). The traditional preparation of Tella from cereals is illustrated in Figure 3 below in a container locally named Roto (Minbale, 2021).

### 3.2 Areki and its production in Ethiopia

*Areki* is a popular liquor in Ethiopia, enjoyed in urban and rural areas. It is a traditional alcoholic beverage distilled from the fermented product (Mulaw et al., 2020). The production process of *Areki* closely resembles that of *Tella*, except for a more concentrated fermentation mass and higher alcohol content. The process involves breaking hot bread into small pieces, mixing it well with water, and allowing four days of fermentation. Subsequently, a part of the mixture is transferred to a traditional distillation device to produce the distilled beverage known as *Areki* (Hotessa & Robe, 2020). The mixture is left to ferment before distillation for 5–6 days.

*Areki*‐tinsis, an *Areki* fermentation product, is created by combining powdered Gesho leaves and powdered malt in a 1 to 2 ratio with water to form a mixture with a free‐flowing consistency. This results in a more concentrated, colorless, and clear traditional alcoholic beverage. *Areki* is more frequently brewed and consumed by farmers and individuals in semi‐urban and rural areas than in urban areas (Belete et al., 2017). It is often consumed by individuals with alcohol dependence who cannot afford factory‐made alcohol. The pH and alcohol content of Ethiopian Areki beverages are explained in Table 2.

**TABLE 2:** pH and alcohol content of Ethiopian Areki beverage.

Collection area	pH	Alcoholic content (% v/v)
Qochi	4.3	36.99
Ajjip	4.4	33.95
Matrik	4.51	39.90
Markato	4.49	38.96
Menihara	4.48	36.30
Average	4.44	37.22

*Note*: Source: Chandravanshi et al. (2017).

### 3.3 *Borde* and *Shamita* beverages

*Borde* and *Shamita* are traditional Ethiopian fermented beverages prepared overnight by LAB for fermentation‐specific cereals, as outlined in Mulaw's work (Mulaw et al., 2019). These beverages, commonly enjoyed in various parts of Ethiopia, particularly the southern region, boast low alcohol content and are nutrient‐rich. *Borde*, in particular, is packed with live cells, enhancing its nutritional value, as indicated in the data presented in Table 3 (Fentie et al., 2020).

**TABLE 3:** Nutrients and ingredients used to prepare *Shamita* and *Borde*.

Beverage	Raw materials	Consumption Places	Nutrients references
*Borde*	Maize, wheat, Finger millet and sorghum	SNNP	Fat (6.88%) Protein (9.5%) Abegaz (2007) Ash (3.66%)
*Shamita*	Barley salt, Linseed, flour, chili pepper	SNNP and A.A	Fat (6.88%) Protein (9.5%) Abawari (2013); Ash (3.66%) Belete et al. (2017)

Source: Fentie et al. (2020).

Abbreviations: A.A, Addis Ababa; SNNP, South nation nationality of people.

Both *Borde* and *Shamita* offer a broad spectrum of carbohydrates and proteins, and their thick consistency makes them a viable staple food, especially for individuals with limited income, reinforcing their significance as a stable food source. The nutrients and ingredients used to prepare *Shamita* and *Borde* beverages, along with their consumption places, shown in Table 3.

#### 3.3.1 Preparation of *Borde*

**FIGURE 4:**
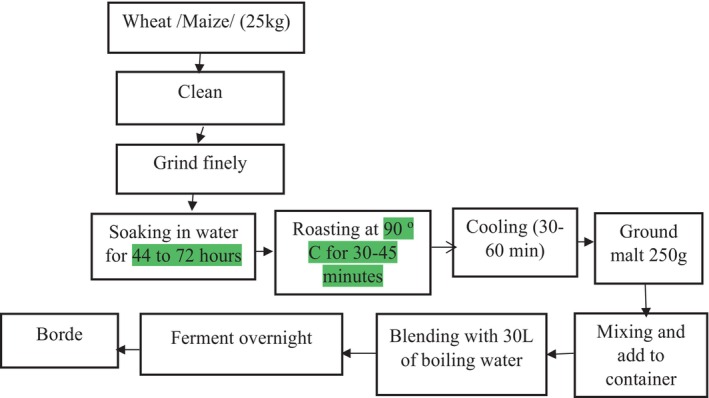
Flow chart of *Borde* preparation.

*Borde*, a widely consumed traditional fermented beverage in Ethiopia, is a common dietary alternative. It is prepared from various cereals and their malt, with maize, barley, and wheat being the primary ingredients. *Borde* holds special significance for lactating mothers, as its consumption post‐childbirth is believed to boost lactation, as highlighted by Kitessa, Bacha, Tola, Murimi, Smith, and Gershe (2022); Kitessa, Bacha, Tola, Murimi, Gershe, and Guta (2022). The preparation of *Borde* involves a series of steps, as illustrated in Figure 4 and detailed in the work of Nemo and Bacha (2020).

#### 3.3.2 Microbiology of *Borde* fermentation

Various microorganisms are involved in the fermentation of *Borde*, as noted by Mulaw et al. (2019). The key fermenting microorganisms are lactic acid bacteria (LAB) and yeast, *Saccharomyces cerevisiae*. Lactic acid bacteria (LAB) are responsible for lactic acid fermentation, while acetic acid fermentation is achieved through the action of Acetobacter bacteria. In Ethiopia, the preparation of fermented beverages is uncomplicated and only requires locally available, inexpensive equipment. The characteristic opaque property of this beverage is attributed to the considerable presence of aerobic mesophilic bacteria, lactic acid bacteria, and yeasts in the malt used during production.

#### 3.3.3 Nutritional value of *Borde* beverage

The nutritional profile of *Borde* reveals the presence of various nutrients, such as protein, fat, ash, and carbohydrates, as detailed in reports by Ashenafi and Mehari (1995) and Fentie et al. (2020). With its modest alcohol content and high carbohydrate concentration, *Borde* is a potent energy source. Furthermore, the lactic acid bacteria and yeasts in *Borde* are recognized as valuable sources of microbial protein due to their high microbial count (Tafere, 2015). Interestingly, *Borde* holds traditional significance for ritualistic and medical purposes, as noted by Tekle et al. (2019), with reported benefits in alleviating issues related to malaria, diarrhea, constipation, and abscesses. Typically made from grains like sorghum, wheat, and maize, *Borde*'s primary ingredients may vary in equal proportions based on the particular recipe.

#### 3.3.4 Quality and safety of fermented *Borde*

*Borde* is known for its short shelf life and develops a sour taste around 16–18 h after preparation. An exemplary *Borde* is described as opaque, fizzy, uniformly turbid, gray in color, and possesses a thick, smooth texture with a flavor profile balancing between sweet and sour (Yugandhar et al., 2021). Due to its single‐protein nature, *Borde* cannot be stored for an extended period. Maintaining the quality and safety of this beverage hinges on the quality of the raw materials and adherence to the Hazard Analysis and Critical Control Points (HACCP) principles. It is advised to consume *Borde* fresh immediately after preparation or store it in cold storage for a brief duration. *Borde* typically contains low alcohol content, with a mean value of around 3.35 ± 0.64 (% v/v), as reported by Nemo and Bacha (2020).

#### 3.3.5 Shamita

*Shamita*, another traditional Ethiopian cereal beverage, is produced by fermenting roasted barley flour overnight and is often consumed as a meal replacement. With a low alcohol content similar to *Borde*, *Shamita* is widely enjoyed across various Ethiopian communities. Wassie and Wassie (2016) noted that *Shamita* is often consumed as a regular food choice due to its thick consistency during financial constraints. The fermentation of *Shamita* primarily involves back slopping, where a small amount of *Shamita* from a previous fermentation is used in combination with the ingredients and tools, leading to the proliferation of lactic acid bacteria and yeast, making it a rich source of microbial protein.

##### 3.3.5.1. Microbiology of *Shamita* fermentation

**FIGURE 5:**
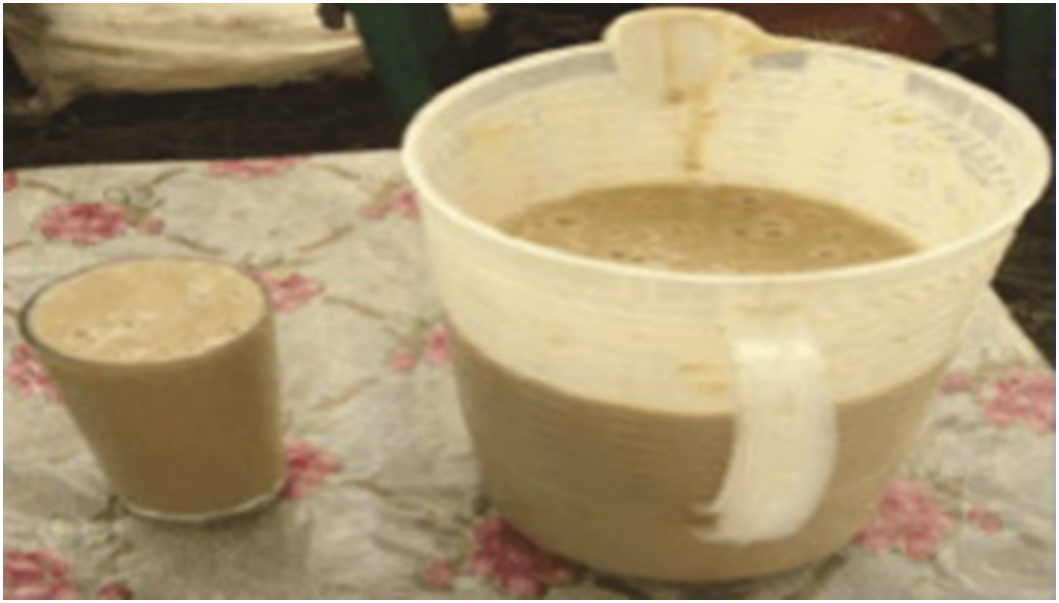
*Shamita* in a glass and jug.

**FIGURE 6:**
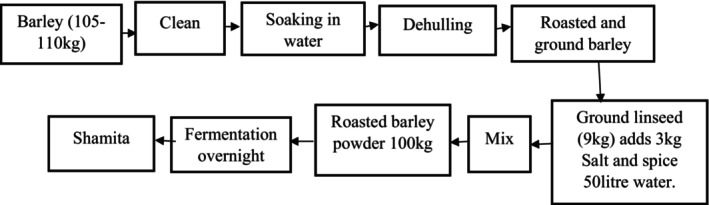
Flow chart of *Shamita* beverage preparation.

Microorganisms responsible for the fermentation of *Shamita* largely stem from back slopping, with live microorganisms contributing to its poor keeping quality and the eventual development of a sour taste due to increased acidity. Like *Borde*, *Shamita's* thick consistency qualifies it as a meal substitute. The preparation of *Shamita* from various ingredients is depicted in Figure 6, and its presentation in a glass and jug is shown in Figure 5 (Lee et al., 2015). As highlighted by numerous researchers, barley serves as a primary source of fermentative microorganisms.

##### 3.3.5.2. Nutritional value of *Shamita*

*Shamita* is typically prepared from maize and wheat. The availability of soluble protein initially increases and then gradually decreases during the preparation process. Often, *Shamita* is made by grinding roasted barley and mixing it with salt, ground linseed, and spices to enhance its flavor. Figure 6 illustrates the steps or flow chart of CCP *Shamita* beverage preparation (Kitessa, Bacha, Tola, Murimi, Gershe, & Guta, 2022; Kitessa, Bacha, Tola, Murimi, Smith, & Gershe, 2022; Wedajo Lemi, 2020). Unlike other varieties, *Shamita* is a fermented porridge with differences in fermentation time, preparation methods, utilization, and ingredient composition. Before being served, *Shamita* is mixed with salt, kochkocha (traditionally milled chili or pepper), and refined butter to enhance its appeal and taste. According to Kitessa, Bacha, Tola, Murimi, Smith, and Gershe (2022); Kitessa, Bacha, Tola, Murimi, Gershe, and Guta (2022), *Shamita* is a significant traditional beverage source of nutritional value, including moisture, crude protein, crude fat, ash content, fiber content, carbohydrates, and energy. Their report particularly suggests that the consumption of this beverage is crucial for expectant and breastfeeding mothers; its preparation is illustrated in Figure 6.

### 3.4 Keribo

**FIGURE 7:**
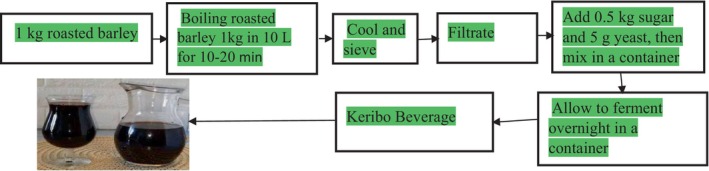
Keribo fermentation process.

*Keribo* is a traditional beverage in Ethiopia, favored by those seeking low‐alcohol drinks with limited financial means (Redeat, 2022). It is widely consumed in Ethiopia's rural and urban areas, particularly in the south, southwest, and eastern regions, where it has deep roots as an ancient fermented beverage. Barley, honey, and sugar are primarily used to produce Keribo. To make Keribo, roasted barley is mixed with hot water. The barley grains are meticulously cleaned before being processed for Keribo. The deeply roasted barley is then added to boiling water, cooled, and sieved. The water‐barley flour mixture is boiled and filtered through a wire‐mesh sieve. Yeast and sugar are added to the filtered mixture in a container. The containers are covered, left to ferment for the next day, and served. Finally, the mixture is given a final addition of sugar before being served to the customer. Figure 7 illustrates the fermentation process of Keribo (Hotessa & Robe, 2020).

Like other beverages, *Keribo* is a fermented product in Ethiopia, created from roasted barley through uncontrolled fermentation and produced on a small scale using locally available equipment. This beverage has a brief shelf life of 2 days when stored at room temperature (Hotessa & Robe, 2020). In Ethiopia, especially in the southwestern region, the fermentation of *Keribo* is intertwined with socio‐cultural practices, mainly due to religious reasons for people who abstain from consuming alcoholic beverages, with *Keribo* serving as a source of energy (Dibaba et al., 2018). Following fermentation, *Keribo* is poured into bottles for sale or consumption, as depicted in Figure 7. It is used for household consumption and also during wedding ceremonies and holidays in various parts of Ethiopia.

### 3.5 Nutritional quality changes through cereal fermentation in traditional beverages

Fermented beverages are prepared globally using diverse production techniques, raw materials, and microorganisms. Fermentation is an age‐old method employed to preserve the nutritional value of beverages by elevating product acidity (Nemo & Bacha, 2020). Recent advancements indicate that fermented beverages are expected to be important in the functional food market (Misihairabgwi & Cheikhyoussef, 2017).

#### 3.5.1 Changes in protein content and quality

Most research indicates that yeast fermentation results in a minor improvement in protein content, chiefly due to the loss in dry matter content, which is primarily composed of carbohydrates. However, significant losses in dry matter were linked to the increase in protein concentration. During fermentation, products' protein content increases due to protein breakdown by microorganisms, leading to the release of peptides and amino acids (Nkhata et al., 2018). The fermentation of pearl millet into fermented pearl millet flour resulted in an increase in protein and amino acids due to the degradation of storage protein and the synthesis of new protein. Fermenting pearl millet with Pleurotus ostreatus led to an increase in amino acid synthesis. Osman (2011) demonstrated that pearl millet fermented for 24 hours experienced an increase in protein due to the loss of carbohydrates.

In a report by Osman (2011), fermenting pearl millet for 24 h amplified the protein content due to the loss of carbohydrates. Post‐fermentation, methionine levels were raised, while lysine, glycine, and arginine were reduced. Although the loss of dry matter during fermentation may contribute partly to the rise in protein, bacterial fermentation is known to enhance the lysine content of fermented grains.

#### 3.5.2 Changes in carbohydrates

Starch and soluble sugars are the primary fermentation ingredients for lactic acid bacteria. Consequently, when grains undergo natural fermentation, a process of depletion occurs, leading to reductions in both the starch content and the overall amount of carbohydrates. During the natural fermentation of sorghum, there was an observed decrease in the starch and fiber contents. At the same time, there was an increase in the concentration of reducing sugars and an enhancement in starch availability.

#### 3.5.3 Changes in lipids

Most research on naturally occurring bacterial or yeast fermentation of grains and legumes has focused on changes in proteins, amino acids, and B‐group vitamins, primarily due to the low‐fat content of cereals. However, such studies are crucial, especially for oil‐rich grains such as corn and pearl millet. Most cereals and legumes are readily available and contain relatively low fat content (Gebretsadik & Negash, 2016). The work of Patel and Matsakas (2019) demonstrated that microorganisms had an excess of lipids and could utilize them to produce phospholipids, acting as emulsifiers, thus leading to changes in the fatty acid profile toward unsaturated fatty acids, which are more abundant in phospholipids during the fermentation process. The presence of lipids in wort and beer is significant due to their impact on yeast metabolism and beer quality (Gordon et al., 2018).

#### 3.5.4 Changes in vitamins

Although there is considerable variation among studies, it has been observed that the vitamin content of cereals, especially the B‐group vitamins, increases during spontaneous fermentation. Fermentation with lactic acid bacteria has increased corn's vitamin B12, folacin, riboflavin, and pantothenic acid natural folic acid concentrations (Levit et al., 2021). Consequently, maize's nutritional value and shelf life are enhanced due to fermentation (Abdul‐Abbas et al., 2022; Kim et al., 2021). The majority of research has highlighted variations in B‐group vitamins during the natural fermentation of grains and grain–legume mixtures, influenced by factors such as the nature of the raw materials, the type and concentration of microflora, temperature, the duration of the fermentation process, and techniques used to measure these vitamins. How these changes in vitamin content operate still needs to be clarified. Thus, more research is necessary (Gebremedhin et al., 2013). The report of Kuyu and Bereka (2019) demonstrated that fermentation preserves the bioactive components of products, such as vitamins, and reduces oxidation, thereby extending the shelf life of products.

#### 3.5.5 Changes in minerals

Cereals are a significant source of dietary minerals, but mineral availability is a critical nutritional concern. The levels of minerals such as calcium (Ca), magnesium (Mg), total phosphorus (P), zinc (Zn), and iron (Fe) did not appear to change significantly. Many researchers noted that fermentation increased the availability of minerals. In finger millet, fermentation was found to be particularly effective at increasing the bioavailability of calcium, phosphorus, and iron. The extractability of minerals significantly increased after the rapid fermentation of barley flour at various temperatures and times (Yugandhar et al., 2021).

### 3.6 The impact of microorganisms on the quality and safety of cereal‐based beverage

The role of microorganisms in shaping cereal‐based beverages' nutritional and safety aspects is significant, as highlighted by the research of Tsafrakidou et al. (2020) and the extensive applications discussed by Gholami‐Shabani et al. (2023). Some of these roles are discussed in the following subsection.

#### 3.6.1 Enhancement of organoleptic properties

Traditional cereal‐based beverages undergo fermentation with microorganisms such as lactic acid bacteria (LAB) and yeast, whose metabolic byproducts contribute to the beverage's acidity, distinctive flavor, and aroma. Microbial fermentation, particularly by lactic acid bacteria, plays a crucial role in maintaining the quality and enhancing the sensory properties of the fermented beverage, thus making it more palatable. As Braide et al. (2018) noted, this process significantly improves the organoleptic characteristics of the beverage. Additionally, the study by Chelule et al. (2010) reported noticeable changes in the organoleptic properties of Tella after a 10‐day fermentation period.

#### 3.6.2 Function as probiotics and enhancement of nutritional quality

Microorganisms in cereal‐based beverages can serve as probiotics, improving nutritional values in food products and promoting better human health.

#### 3.6.3 Bio‐preservative properties

Microorganisms contribute to bio‐preservation, leveraging natural antimicrobials to extend the shelf life of food products. Many bacteria involved in the fermentation of foods produce bioactive molecules such as hydrogen peroxide, organic acids, and bacteriocins, which act as potent bio‐preservatives (Amiri et al., 2021). The acid content in alcoholic beverages plays a crucial role in maintaining freshness and extending shelf life by inhibiting the growth of pathogenic microbes. LAB, for instance, exhibits antifungal properties, contributing to the prolonged shelf life of fermented foods by preventing spoilage. This bio‐preservative action helps ensure that fermented foods maintain quality over time.

### 3.7 The role of traditionally fermented beverages in food systems

Malnutrition and nutritional deficiencies remain pervasive challenges in developing and underdeveloped nations. Poor diet, inadequate food intake, reduced nutrient bioavailability, and the prevalence of infectious diseases are widely considered the primary causes of malnutrition. Moreover, concerns regarding microbial contamination and naturally occurring toxins are paramount to ensuring food safety. Simple, cost‐effective, and traditional food‐based solutions, such as the fermentation of beverages, are believed to offer effective remedies for these issues. Fermentation diminishes antinutritional factors, prolongs the product's shelf life and safety, and enhances its nutritional quality, digestibility, and nutrient bioavailability (Tamang et al., 2020).

Current efforts to alleviate hunger encompass increased agricultural production, raw material processing, nutritional supplementation, and food fortification. Locally grown foods play a critical role in food systems by combating hunger and malnutrition (Materia et al., 2021). Fermentation, an age‐old processing technique, leverages microbial activity to transform raw materials and is traditionally overseen by women.

The food systems approach has garnered significant attention in endeavors to eradicate hunger. Despite these efforts, global food security remains tenuous, posing challenges to delivering nutritious food to the world's population.

Local foods play a significant role in the diets of local populations; however, this significance has yet to be duly reflected in the focus on local beverages in extensive initiatives aimed at ending hunger, advancing nutrition, and improving living conditions. Traditional fermented beverages hold a special place among processed local foods, as they have historically played a significant role in human diets across virtually all cultures and continents since the dawn of civilization (Tamang et al., 2020).

In most cases, locally available raw materials are utilized for the homemade processing of traditional fermented beverages, primarily for household consumption. Some processors may distribute their surplus products in local markets, while a smaller number may specifically engage in production for commercial sale. While some fermented foods contain alcohol, the majority are acidic. The pre‐processing of raw materials typically constitutes the initial phase of production. One primary advantage of fermentation is the removal of food‐borne pathogens from raw materials due to increased acidity and/or the presence of alcohol after fermentation. This process also enhances the shelf life and the commercial value of the raw materials, providing producers with opportunities to improve their living standards through sales (Chileshe, van den Heuvel, et al., 2020).

Furthermore, fermentation's additional benefit lies in improving the availability of food components in the human digestive tract, thereby enhancing the nutritional value of the raw material. For example, the process can enable the pre‐digestion of complex carbohydrates and enhance the bioavailability of essential micronutrients such as iron and zinc (Marco et al., 2017).

### 3.8 Entrepreneurial opportunities in processing fermented foods

Small‐scale fermentation activities hold the inherent potential to positively impact food security and economic development by reducing post‐harvest food losses and increasing the availability of nutritious and appealing foods. Fermentation‐related endeavors significantly contribute to entrepreneurship in rural areas, where most of the raw materials for fermentation are cultivated. This fosters employment opportunities, curbs rural–urban migration, and addresses associated social issues (Kadirvel et al., 2018).

### 3.9 Socio‐cultural significance of Ethiopian cereal‐fermented beverages

Ethiopian cereal‐fermented beverages are essential regarding nutrition, socio‐cultural, and economic aspects. They play a crucial socio‐cultural and economic role in Ethiopian society, deeply embedded in local culture. Frequently associated with traditions of hospitality and camaraderie, these beverages are an integral part of social customs within most families. They serve as a unifying element, fostering cordial relationships. Traditional Ethiopian beverages and funerals are typically consumed during fieldwork and festive occasions (e.g., weddings, naming ceremonies, and initiation rites). These fermented drinks function as dietary supplements, akin to using weaning foods to complement breastfeeding. The quintessential setting for consumption is the communal gathering, where a sense of conviviality, sociability, and social connection shapes the perception of the product's quality and consumption patterns.

### 3.10 Safety attributes of traditional fermented foods

Fermentations that involve the generation of lactic acid are typically benign. In these processes, fermentable sugars are converted to lactic acid. Maintaining the optimal fermentation temperature promotes beneficial fermentation organisms' growth while inhibiting spoilage organisms' proliferation. Given that many fermented beverages are produced using microorganisms, there is a significant risk of toxin contamination. Therefore, the proper application of soaking is crucial for ensuring the safety of cereal‐fermented beverages (Chileshe, Talsma, et al., 2020). Fermented beverages are produced worldwide using various manufacturing techniques, raw materials, and microorganisms. Fermentation is one of the old methods used to preserve the nutritional value of beverages by increasing the acidity of products (Nemo & Bacha, 2020). Based on recent developments, it is anticipated that fermented beverages will continue to be a significant component of the functional food market (Misihairabgwi & Cheikhyoussef, 2017).

## 4 CONCLUSION

Cereal‐based traditional fermented beverages play a significant role in food systems, serving as a crucial preservation method. In Ethiopia, alcoholic beverages such as *Tella*, *Borde*, *Shamita*, *Areki*, and *Keribo* are integral to various ceremonies, weddings, festivals, and social gatherings. Their contribution to traditional fermented beverages helps alleviate food system issues while offering nutritional and health‐promoting benefits. To ensure the quality and safety of these beverages, it is essential to employ proper production methods and adhere to Hazard Analysis Critical Control Point (HACCP) principles. Ethiopian cereal beverages' production, quality, and safety hinge on the raw materials used and the processing methods employed.

Developing standardized procedures for the processing of traditional beverages at the local level, particularly considering factors such as the influence of high temperatures, ingredient proportions, and fermentation duration, is crucial for designing and optimizing the quality of these beverages. Furthermore, scientific investigations need to focus more on the nutritional, antinutritional, and other health‐promoting components of all traditional cereal‐based beverages. Additionally, essential process parameters for producing safe and higher‐quality traditional cereal beverages have not been scientifically optimized and established locally.

## AUTHOR CONTRIBUTIONS

The first author was responsible for searching, looking at literature data, and writing up a paper. The second author contributed to manuscript preparation and standardized the paper by making edits across the whole document. Both authors equally contributed to the preparation of the manuscript and approved the final manuscript for publication.

## ETHICS STATEMENT

This study does not involve any human or animal testing.

## Supporting information


Figure S1.

## Data Availability

All relevant data for the work are included in the manuscript.
