# Supplementary figures and images for: A review of the production, quality, and safety of traditionally fermented cereal‐based alcoholic beverages in Ethiopia
Source: Food Sci Nutr. 2024 Feb 13;12(5):3125–36. doi: 10.1002/fsn3.4012 (PMC11077225; doi:10.1002/fsn3.4012)

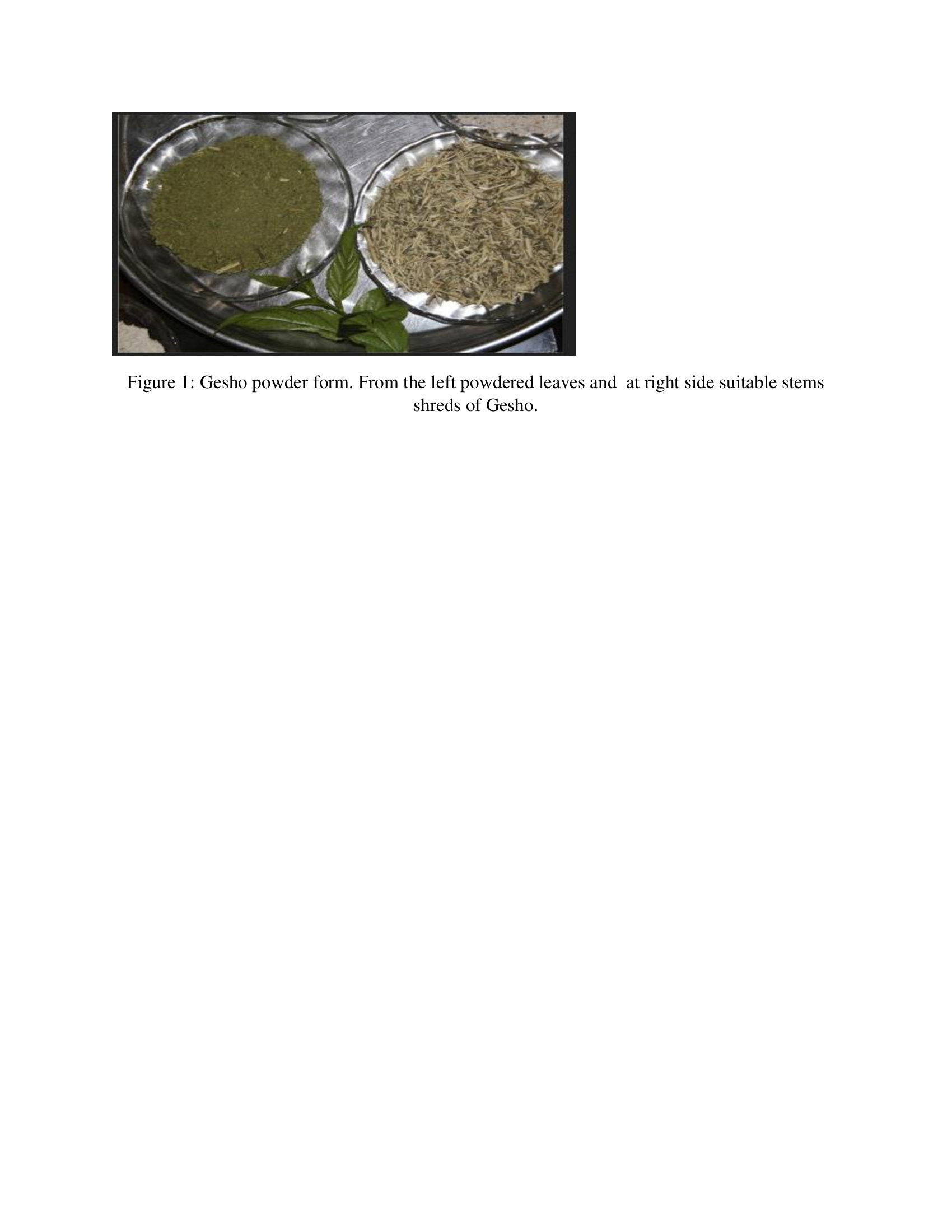

Supplement: Supplementary file 1 — Figure S1. [file FSN3-12-3125-s001.jpg]
